# Treatment of Large Liver Cyst Evaluated with CA19-9 in the Cystic Fluid

**DOI:** 10.1155/1996/34658

**Published:** 1996

**Authors:** Yasuharu Ikeda, Katsumi Kawasaki, Tetsuo Ikeda, Hiroyuki Uchida, Kazumasa Morinaga

**Affiliations:** 1 Department of Surgery Hahnemann University Broad & Vine Philadelphia PA 19102-1192 USA; 2 The Department of Surgery Saiseikai-Karatsu Hospital Saga Japan

## Abstract

A middle-aged woman was admitted with a diagnosis of liver cysts. The patient was symptomatic and was treated by injecting absolute ethanol into the largest cyst every week, but the secretion from the cyst persisted. The patient was then treated by absolute ethanol injection every day with good results. CA 19-9 was measured in the cystic fluid. The secretion was related to CA 19-9 activity.

Thus, for patients with symptoms from a liver cyst, the injection of ethanol every day can be effective. CA19-9 level in the contents of the liver cyst was an important factor in assessing the effect of ethanol injection on the liver cyst.


*HPB Surgery*, 1996, Vol.9, pp.179–182
Reprints available directly from the publisher
Photocopying permitted by license only

© 1996 OPA (Overseas Publishers Association)
Amsterdam B.V. Published in The Netherlands
by Harwood Academic Publishers GmbH
Printed in Malaysia

### CASE REPORT

### Treatment of Large Liver Cyst Evaluated with CA19-9 in the Cystic Fluid

YASUHARU IKEDA*, KATSUMI KAWASAKI, TETSUO IKEDA, HIROYUKI UCHIDA and KAZUMASA MORINAGA

The Department of Surgery, Saiseikai-Karatsu Hospital, Saga, Japan

*(Received 29 October 1994)*

A middle-aged woman was admitted with a diagnosis of liver cysts. The patient was symptomatic and was treated by injecting absolute ethanol into the largest cyst every week, but the secretion from the cyst persisted. The patient was then treated by absolute ethanol injection every day with good results. CA 19-9 was measured in the cystic fluid. The secretion was related to CA 19-9 activity.

Thus, for patients with symptoms from a liver cyst, the injection of ethanol every day can be effective. CA19-9 level in the contents of the liver cyst was an important factor in assessing the effect of ethanol injection on the liver cyst.

KEY WORDS: Liver cyst ethanol injection CA 19-9

### INTRODUCTION

Nonparasitic liver cyst, once considered to be a rare occurrence is now more often detected during clinical work-up which includes use of ultrasonography (US) and computed tomography (CT). Although commonly asymptomatic, these cysts may be accompanied by symptoms of discomfort, abdominal mass, hepatomegaly and abdominal pain[1] and there is a risk of rupture[2] should the cyst becomes enlarged. Resection[3], deroofing[4] and fenestration[5] can be performed to treat these non-parasitic liver cysts. Bean and co-workers[6,7] reported that the direct injection of alcohol into renal and liver cysts was an effective treatment. We describe here our findings and treatment of a woman with symptomatic liver cyst.

### CASE REPORT

Case: A 72-year-old woman admitted to Saiseikai-Karatsu Hospital in May 1992 with complaints of an abdominal mass and general fatigue. On physical examination she was alert, temperature was 35.8°C, anemia or jaundice was absent and abdominal examination revealed a mild abdominal distention with 10 cm palpable liver in the epigastrium. Laboratory findings on admission were as follows: The red cell count was 446 × $10^4$/mm$^3$; the hematocrit was 43.2 %; the hemoglobin was 14.7 g/dl; the white cell count was 5900 /mm$^3$; the platelet count was 14.3 × $10^4$ /mm$^3$. The totalprotein was 85 g/l.Urea nitrogen was 16.4µmol/1 the creatinine 62 µmol/l, the bilirubin was 170 µmol/l, glutamate oxaloacetate transaminase (GOT) 23 IU/L, glutamate pyruvate transaminase (GPT) 17 IU/L, lactate dehydrogenase (LDH) 537 IU/L, alkaline phosphatase (ALP) 376 IU/L, r-glutamyl transpeptidase (r-GTP) 18 IU/L, alpha fetoprotein 10 ng/ml and CA19-9 39.4 U/ml. Chest x-ray films and electrocardiograms revealed no abnormalities. Abdominal CT and US showed multiple cysts in the liver (Fig. 1). Drip infusion cholangiography was performed before CT to test for communication between the liver cyst and the biliary trees or vessels (DIC-CT). Skin and antibody tests for parasitic disease were negative. Cytologic and bacteriologic evaluations were also negative for malignancy or infection.

**Correspondence to*: Yasuharu Ikeda MD, Department of Surgery, Hahnemann University, Broad & Vine, Philadelphia, PA 19102-1192 U.S.A.

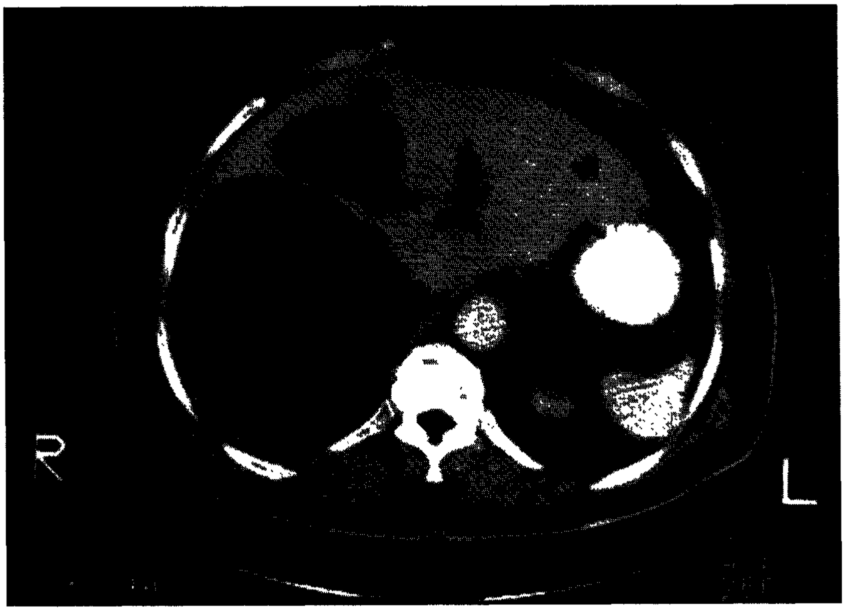

**Figure 1** CT showed giant liver cyst before treatment

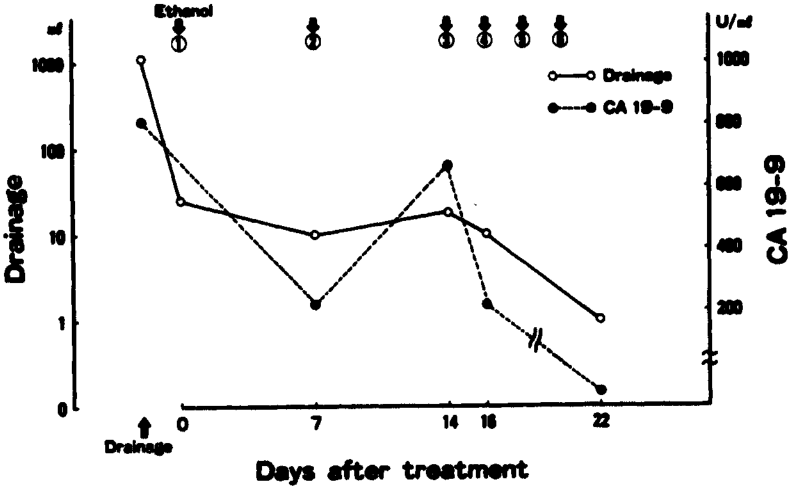

**Figure 2** Secretion volume and CA 19-9 of secretion from liver cyst after ethanol injection

The patient underwent perctaneous transhepatic drainage of the largest cyst with US and absolute ethanol injection into the largest cyst every week. The cyst transiently decreased after these treatments but the secretion from cyst persisted. The patient was then treated by absolute ethanol injection every day with good results. The clinical status and the CT showed a decrease in the size of the cyst. Secretion volume and CA 19-9 level in the secretion from the liver cyst after ethanol injection were shown in Figure 2. Before ethanol injection, CA 19-9 in the cystic fluid was 804 U/ml. At the end of ethanol injection treatments, CA 19-9 was decreased to 21 U/ml and secretion from the cyst was less than 10 ml/day. She was discharged on the 29th Post-treatment day and is doing well at present. Twelve months after treatment, CT showed the cyst had disappeared without recurrence (Fig. 3).

### DISCUSSION

The classification of liver cysts as proposed by Henson *et al.*[8] was based essentially on congenital, traumatic, inflammatory and neoplastic origins. With the exten-

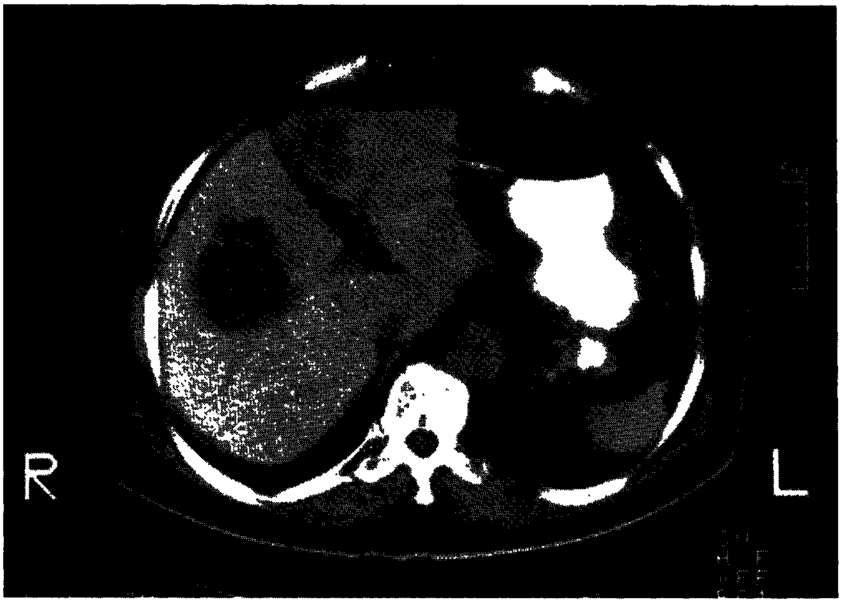

**Figure 3** Six months after treatment, CT showed the cyst had disappeared without recurrence.

sive application of US and CT, asymptomatic congenital hepatic cysts are being increasingly identified[9,10]. Most patients have congenital hepatic cysts which are small and asymptomatic, but occasionally large cysts may form a mass in the upper abdomen and vague gastrointestinal discomfort ensues. Asymptomatic liver cysts do not require treatment, when there is no evidence of a malignancy or when spontaneous rupture of the liver cyst does not occur. The occurence of carcinoma in solitary non-parasitic cysts of the liver is rare[11], as is of spontaneous rupture of cysts[12].

Adequate methods of treating symptomatic non-parasitic liver cysts, include simple aspiration[13], resection, marspialization, external drainage into a jejunal loop[14], deroofing, fenestration, resection-fenestration [15] and complete excision of the cysts. In our patients, a communication between the cyst and the biliary system was not confirmed.

Andersson *et al.*[16] reported that nine patients with hepatic cysts were effectively treated with puncture, drainage and injection of alcohol into the cyst. We reported that when a malignancy was suspected at the time of cytologic and imaging studies, or when alcohol administration was ineffective, then surgery should be considered[17,18].

Carbohydrate antigen (CA19-9) is a tumor-associated antigen which shows a high sensitivity for cancer, and is now widely used as a tumor marker in the diagnosis of this condition[19-23]. Atkinson *et al.*[19] reported that CA19-9 has been shown to be produced by human tumor and normal tissues. In this case, there was the relation between the secretion volume and CA19-9 level in the secretion from the liver cyst after ethanol injection.

In conclusion, ethanol injection into the cyst every day was effective and CA19 - 9 level in the cystic fluids was an important factor in measuring the effect of ethanol injection with liver cyst.

*Acknowledgments*

We thank Brenda Norris for comments on the manuscript.

### REFERENCES

1. Sanfelippo P.M, Beahrs O.H, Weiland L.H. (1974) Cystic disease of the liver. *Ann Surg* : **179**: 922–925.
2. Hadad A.R, Westbrook K.C, Graham G.G. *et al.* (1977) Symptomatic nonparasitic liver cysts. *Am J Surg* **134**: 739–744.
3. Armitage N.C, Blumgart L.H.( 1984) Partial resection and fenestration in the treatment of polycystic liver disease. *Br J Surg* **71**: 242–244.
4. Belcher H.V, Hull H.C. (1969) Nonparasitic cysts of the liver: Report of three cases. *Surgery* **65**: 427–431.
5. Lin T.Y, Chen C.C, Wang S.M. (1968) Treatment of non-parasitic cystic disease of the liver: a new approach to therapy with polycystic liver. *Ann Surg* **168**: 921–927.
6. Bean W.J. (1981) Renal cysts: treatment with alcohol. *Radiology* **138**: 329–331.
7. Bean W.J, Rodan B.A. (1985) Hepatic cysts: treatment with alcohol. *Am J Roent* **144**: 237–241.

8. Henson S.W, Grey H.K. (1957) Dockerty MB. Benign tumors of the liver, IV. Polycystic disease of surgical significance. *Surg Gyneco Obstet* **104**: 63–67.
9. Solgaard S, Burcharth F Miskowiak J. *et al.* (1984) Ultrasonically diagnosed simple cysts of the liver. *Radiologie* **24**: 195–197.
10. El-Tahir M.I, Omojola M.F, Malatani T. *et al.* (1992) Hydatid disease of liver: evaluation of ultrasound and computed tomography. *Br J Radiol* **65**: 390–392.
11. Bloustein P.A. (1977) Association of carcinoma with congenital cystic conditions of the liver and bile ducts. *Am J Gastroenterol* **67**: 40–46.
12. Morgenstern L. (1959) Rupture of solitary nonparasitic cysts of the liver. *Ann Surg* **150**: 167–171.
13. Flagg R.S, Robinson D.W. (1967) Solitary nonparasitic hepatic cysts: report of oldest known case and review of the literature. *Arch Surg* **95**: 964–973.
14. Longmire W.P, Mandiola S.A. Gordon H.E. (1971) Congenital cystic disease of the liver and biliary system. *Ann Surg* **174**: 711–726.
15. Newman K.D, Torres V.E, Rakela J. *et al.* (1990) Treatment of highly symptomatic polycystic liver disease. *Ann Surg* **212**: 30–37.
16. Andersson R, Jeppsson B, Lunderquist A. *et al.* (1989) Alcohol sclerotherapy of non-parasitic cysts of the liver. *Br J Surg* **76**: 254–255.
17. Furuta T, Yoshida Y, Saku M. *et al.*(1990) Treatment of symptomatic non-parasitic liver cysts. *HPB Surg* **2**: 269–279.
18. Ikeda Y, Kanematsu T, Matsumata T. *et al.* (1992) Symptomatic polycystic liver disease and surgical treatments. *Hepatogastroentelol* **39**: 227–230.
19. Atkinson B.F, Ernst C.S. Herlyn M. *et al.* (1982) Gastroin testinal cancer-associated antigen in immunoperoxidase aassay. *Cancer Res* **42**: 4829–4823
20. Del Villano B.C, Brennan S, Brock P. *et al.* (1983) Radioimmunometric assay for a monoclonal antibody-define tumor marker, CA 19-9. *Clin Chem* **29**: 549–552.
21. Gupta M.K, Arciaga R, Bocci L. *et al.* (1985) Measurement of a monoclonal-antibody-defined antigen (CA 19-9) in the sera of patients with malignant and nonmalignant disease. *Cancer* **56**: 277–283.
22. Andriulli A, Gindro T, Piantino P. *et al.* (1986) Prospective evaluation of the diagnostic efficiency of CA 19-9 assay as a marker for gastrointestinal cancers. *Digestion* **33**: 26–33.
23. Sakamoto K, Haga Y, Yoshimura R. *et al.* (1987) Comparative effectiveness of tumour diagnostics, CA 19-9, CA 125 and carcinoembryonic antigen in patients with diseases of the digestive system. *Gut* **28**: 323–329.

